# Study on the Efficacy and Mechanism of *Lycium barbarum* Polysaccharide against Lead-Induced Renal Injury in Mice

**DOI:** 10.3390/nu13092945

**Published:** 2021-08-25

**Authors:** Wen Xie, Yuan-Yuan Huang, Hua-Guo Chen, Xin Zhou

**Affiliations:** 1 Key Laboratory for Information System of Mountainous Areas and Protection of Ecological Environment, Guizhou Normal University, 116 Baoshan North Rd., Guiyang 550001, China; 19010160373@gznu.edu.cn (W.X.); 20010160452@gznu.edu.cn (Y.-Y.H.); 2 Guizhou Engineering Laboratory for Quality Control & Evaluation Technology of Medicine, 116 Baoshan North Rd., Guiyang 550001, China; 3 The Research Center for Quality Control of Natural Medicine, Guizhou Normal University, 116 Baoshan North Rd., Guiyang 550001, China

**Keywords:** *Lycium barbarum* polysaccharide, lead-induced renal injury, oxidative stress, inflammatory, apoptosis, Nrf2 signaling pathway

## Abstract

Lead is one of the most common heavy metal pollutants in the environment. Prolonged exposure to lead will induce oxidative stress, inflammation, and apoptosis in the kidneys, which in turn causes kidney injury. *Lycium barbarum* polysaccharide (LBP) is well known for its numerous pharmacological properties. This study aims to explore the efficacy and mechanism of LBP against lead-induced kidney damage in mice. Symptoms of renal injury were induced in mice by using 25 mg/kg lead acetate (PbAc_2_), and different doses of LBP (200, 400, and 600 mg/kg BW) were orally administrated to PbAc_2_-treated mice for five weeks. The results of the pharmacodynamics experiment showed that the renal pathological damages, serum creatinine (Cre), blood urea nitrogen (BUN), and kidney index of PbAc_2_-treated mice could be significantly alleviated by treatment with LBP. Further, LBP treatment significantly increased the weight and feed intake of PbAc_2_-treated mice. The dose effect results indicated that a medium dose of LBP was superior to high and low doses. The results of mechanistic experiments showed that LBP could attenuate oxidative stress, inflammation, and apoptosis in the kidneys of mice with lead toxicity by activating the nuclear factor erythroid 2-related factor 2 (Nrf2) signaling pathway.

## 1. Introduction

Lead is considered one of the most polluting elements in the world and carries high non-carcinogenic risks for children and adults [1,2]. Lead pollution in the environment mainly comes from lead–zinc ore smelting, non-ferrous metal polymetallic ore smelting, electronic waste recovery, and leaded gasoline consumption [3]. Lead can be absorbed by humans via the alimentary tract, respiratory tract, and skin [4]. After intake of lead, it firstly enters the bloodstream, and during circulation, toxic metals are distributed to the kidneys, causing kidney damage [5].

The mechanisms underlying renal injury due to lead toxicity are multifaceted. Studies have shown that lead exposure induces renal inflammation and increases the levels of interleukin-1β (IL-1β), tumor necrosis factor-α (TNF-α), and nitric oxide (NO) in the kidney tissue [6]. Furthermore, lead exposure can promote an oxidative stress response, significantly increase the level of malondialdehyde (MDA) in renal tissue, and reduce the levels of superoxide dismutase (SOD), glutathione (GSH), and glutathione S-transferase (GST) in the tissue, which in turn induces the excessive production of reactive oxygen species (ROS) and stimulates an apoptotic cascade reaction, leading to the release of cytochrome-C and the activation of the caspase (Casp) enzyme. At the same time, it significantly increases the level of Bax in the renal tissue and reduces the level of Bcl-2, to induce apoptosis in the renal tissue [7,8,9,10,11,12].

To date, chelation is still a well-known treatment for heavy metal poisoning. Chelating agents can combine with metal ions to form a complex ring-like structure to enhance their clearance from the body [13]. For example, 2,3-dimercaptopropanol has long been the mainstay of chelation therapy for lead poisoning; hydrophilic chelating agents such as meso-2,3-dimercaptosuccinic acid can effectively promote the excretion of metal in the kidney [14]. Paradoxically, metal chelate agents have disadvantages such as neurotoxicity, hepatotoxicity, loss of essential metal elements (Zn, Cu), and some serious mucocutaneous reactions [15]. Therefore, it is of great theoretical value and practical significance to seek effective and low-side-effect drugs to protect against lead-induced renal injury.

In recent years, natural alternatives have attracted much attention due to their affordability, availability, and minimal side effects [13,16,17,18]. *Lycium barbarum* L. is a traditional medicinal and edible plant in China [19]. *Lycium barbarum* polysaccharide (LBP) is one of the main active ingredients in *Lycium barbarum* L. It has been studies in terms of food, medicine, and other research fields due to its multiple biological functions and advantages such as low cost and good availability [20,21,22,23,24,25,26,27,28,29]. However, the efficacy and mechanism of LBP against lead-induced kidney injury have not been investigated to date.

In this study, the lead acetate (PbAc_2_) was used to establish a kidney injury model, and different doses of LBP (200, 400, and 600 mg/kg) were administered to mice with lead toxicity to explore the dose–effect relationship of LBP in resisting lead-induced kidney injury. Further, the pharmacodynamics were evaluated based on the mouse body weight, feed intake, kidney index, renal function markers (serum creatinine, Cre; blood urea nitrogen, BUN), and pathological sections of kidney tissues. Finally, the detection of oxidative stress markers (SOD, MDA, GST, and GSH), inflammatory markers (IL-1β, TNF-α, and NO), apoptosis markers (Bax, Bcl-2, and Casp-3), and nuclear factor erythroid 2-related factor 2 (Nrf2) signaling pathway-related proteins (oxygenase-1, HO-1; Kelch-like ECH-associated protein 1, Keap1; NAD(P)H dehydrogenase, quinone 1, NQO1) was used to investigate the mechanism. It is hoped that this study will provide useful information for the development of LBP-based nutraceuticals and auxiliary drugs.

## 2. Materials and Methods

### 2.1. Materials

LBP (purity ≥ 90%, batch number: BCSW200910-3) was bought from BaiChuanKangZe Biology Science and Technology Co., Ltd. (Xi’an, Shaanxi, China). Lead acetate (batch number: HG/T2630-2010) was purchased from KeMiOu Chemical Reagent Co., Ltd. (Tianjin, China). Mouse BUN, Cre, GST, SOD, GSH, MDA, IL-1β, TNF-α, NO, Casp-3, Bax, and Bcl-2 ELISA kits were purchased from Shanghai Qiaoyu Biotechnology Co., Ltd. (Shanghai, China). Anti-NQO1 and anti-heme oxygenase-1 (HO-1) antibodies were purchased from Abcam (Shanghai, China). Nrf2 and Keap1 antibodies were purchased from Cell Signaling TECHNOLOGY (Shanghai, China). All other chemicals and reagents were of analytical grade.

### 2.2. Animals and Experimental Design

Male ICR mice (4–6 weeks old, 18–22 g) were used for this study. The animals were provided by Changsha Tianqin Biotechnology Co., Ltd. (Hunan, China, License number: SCXK(X) 2019-0013). All of the mice were housed under controlled conditions at 22 ± 2 °C, with a relative humidity of 60 ± 10%, and in an alternating 12 h light–dark cycle. The mice were provided with a standard laboratory diet and water. The mice were cared for and treated under the guidelines established by the Chinese Council on Animal Care, as approved by the Guizhou Normal University Animal Care and Use Committee.

The dose and method of administration were chosen based on previous literature with modifications [6,30,31]. After three days of adaptive feeding, 50 mice were randomly divided into five groups, with ten mice per group (*n* = 10) as follows:

Group 1 (Control group): Mice were intragastrically administered with 0.1 mL/10 g normal saline twice a day, with an interval of 2 h, for 35 consecutive days.

Group 2 (Model group): Mice were intragastrically administered with 25 mg/kg of lead acetate solution (the solution was freshly prepared before use), and after 2 h, the mice were intragastrically administered with 0.1 mL/10 g of normal saline, once daily for 35 consecutive days.

Group 3 (Low-dose group): Mice were intragastrically administered with 25 mg/kg of lead acetate solution, and after 2 h, mice were intragastrically administered with 200 mg/kg of LBP, once daily for 35 consecutive days.

Group 4 (Medium-dose group): Mice were intragastrically administered with 25 mg/kg of lead acetate solution, and after 2 h, mice were intragastrically administered with 400 mg/kg of LBP, once daily for 35 consecutive days.

Group 5 (High-dose group): Mice were intragastrically administered with 25 mg/kg of lead acetate solution, and after 2 h, the mice were intragastrically administered with 600 mg/kg of LBP, once daily for 35 consecutive days.

The administration time of each group of mice was roughly the same, and the body weight and feed intake of the mice were recorded during the experiment. The mice fasted for 12 h on day 35, and their blood and kidneys were collected on day 36.

### 2.3. Blood and Kidney Tissue Collection

The mice were anesthetized, and blood was collected orbitally in 1.5 mL, clean sterile centrifuge tubes, then immediately centrifuged (3000 r/min, 10 min) before serum was collected and stored in a −20 °C freezer for later use. The mice were sacrificed by cervical dislocation; the kidneys were then immediately removed, and the bloodstain was rinsed with pre-cooled saline. After blotting on filter paper, the mass was weighed with a precision electronic balance; the left kidney was immediately immersed in the configured formalin solution for further use, and the right kidney was preserved in a −80 °C freezer for further use.

### 2.4. Study on the Pharmacodynamics of LBP against Lead-Induced Renal Injury in Mice

#### 2.4.1. Measurement of Body Weight of Mice and Daily Feed Intake

The mice were weighed every seven days, and the average weight of the mice in each group was calculated. Each group was fed with 70 g feed every day (making sure there was a daily surplus of feed), and the remaining feed was weighed at 9 am the next day to calculate the daily feed intake of each group.

#### 2.4.2. Measurement of Renal Index

The kidney was weighed, using the following formula: Organ weight index = [Wet weight of organ (g)/Bodyweight (g)] × 100.

#### 2.4.3. Detection of Renal Function Biomarkers

After thawing the serum, the expression levels of Cre and BUN in the mouse serum were measured by ELISA. After sequential addition of samples, standards, antibodies, and color development agents according to the procedure of the kit, the concentrations of Cre and BUN in the mouse serum were calculated by measuring the absorbance (OD value) at a 450 nm wavelength using a microplate reader (model number: Max Plus 384).

#### 2.4.4. Histopathological Examination

Formalin solution (10% formaldehyde in distilled water) was used to fix the kidney samples paraffin embedding. The kidney samples were stained with hematoxylin-eosin (H&E) and 4 µm sections were cut for histopathological evaluations. Images were captured at a 400 times magnification. Pathological changes of the glomerulus, renal tubular injury, inflammation were graded as mild (+), moderate (++), or severe (+++) in each group.

### 2.5. Study on the Mechanism of LBP against Lead-Induced Renal Injury in Mice

#### 2.5.1. Detection of Oxidative Stress Markers in Renal Tissue

We took 0.1 g renal tissue, added 0.9 mL normal saline, made a 10% homogenate of the renal tissue, centrifuged (3000 r/min, 10 min), and took the supernatant. The expression levels of SOD, MDA, GSH, and GST were detected by ELISA. After sequential addition of samples, standards, antibodies, and color development agents according to the procedure of the kit, the concentrations of SOD, MDA, GSH, and GST in the mouse serum were calculated by measuring the absorbance (OD value) at a 450 nm wavelength using a microplate reader.

#### 2.5.2. Detection of Renal Inflammatory Biomarkers

The IL-1β, TNF-α, and NO levels in the renal tissue were determined using ELISA kits according to the manufacturer’s guidelines (consistent with 2.5.1 operation steps).

#### 2.5.3. Detection of Apoptotic Biomarkers

The Cas-3, Bax, and Bcl-2 levels in the renal tissue were determined using ELISA kits according to the manufacturer’s guidelines (consistent with 2.5.1 operation steps).

#### 2.5.4. Western Blotting Analyses

After thawing the kidney tissue of each group of mice, 40 mg of homogenate was taken, and the total protein was extracted with the BCA kit to determine the protein content. We extracted the same amount of protein sample and denatured it at 100 °C for 5 min. We used the sodium lauryl sulfate-polyacrylamide gel electrophoresis method to separate the protein and transfer it to a polyvinylidene fluoride membrane, where we added the corresponding primary antibody at 4 °C and incubated overnight, before washing and adding horseradish peroxide at 4 °C. The enzyme-labeled secondary antibody was incubated for 2 h; then, we added luminescent solution after washing, and exposed [11]. We used ImageJ to count the gray values.

### 2.6. Statistical Analysis

Data analysis was carried out using the Statistical Package for Social Sciences (SPSS version 26) software. The collected data were analyzed using one-way analysis of variance (ANOVA). A value of *p* < 0.05 was considered statistically significant. All values were expressed as the mean ± standard deviation (SD) of the mean.

### 2.7. Dose–Effect Analysis

Orthogonal Partial least squares discriminant analysis (OPLS-DA) is often used to deal with classification and discriminant problems. It was similar to principal component analysis (PCA) except that PCA was unsupervised and OPLS-DA was supervised [32]. In order to visualize the dose–effect relationship of LBP against lead-induced renal injury, SIMCA-P14.1 software was used for multivariate statistical analysis. The expression levels of oxidative stress markers (SOD, MDA, GSH, GST), inflammatory markers (TNF-α, IL-1β, NO), and apoptotic markers (Casp-3, Bax, Bcl-2) were used as variables to establish an unsupervised PCA model and a supervised OPLS-DA model. Finally, Oebiotech software (available online: https://cloud.oebiotech.cn/task/website (accessed on 16 July 2021)) was used to analyze the efficacy index and mechanism index by cluster heat map, and the global expression change of multisample and multivariable is presented intuitively.

## 3. Results

### 3.1. Effect of LBP on Lead-Induced Kidney Injury in Mice

#### 3.1.1. Effect of LBP on Body Weight

**Figure 1:**
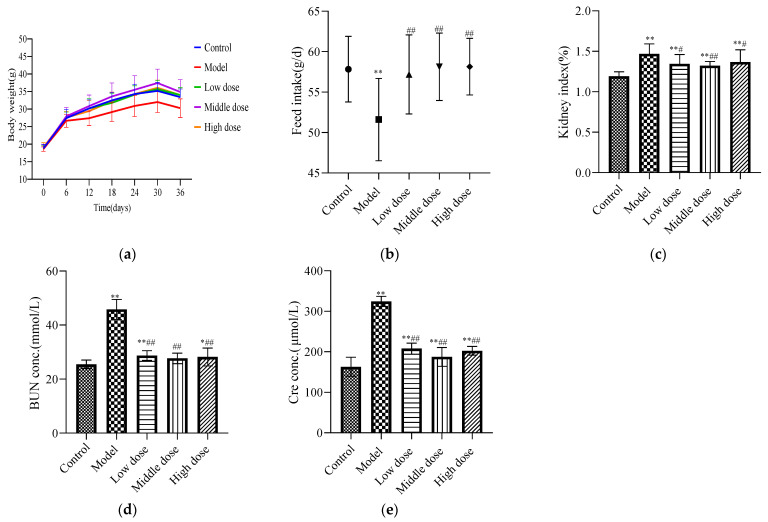
Pharmacodynamic evaluation index for LBP against lead-induced renal injury. The results are expressed as the mean ± SD. * Statistically significant compared to control group, * *p* < 0.05, ** *p* < 0.01. ^#^ Statistically significant compared to model group, ^#^ *p* < 0.05, ^##^ *p* < 0.01. (**a**) Weight change of mice; (**b**) feed intake of mice; (**c**) renal index; (**d**) expression level of BUN; (**e**) expression level of Cre.

There was no significant difference in the initial body weights of the mice in each group (Table 1). During the experiment, the model group had the slowest growth rate, the middle-dose group had the fastest growth rate, and the high-dose, low-dose, and control groups had no significant differences. After the experiment, the average body weight of mice in the model group was significantly lower than that of the control group (*p* < 0.05), while that of the LBP administration group was significantly higher than that of the model group (*p* < 0.01) with no significant difference from the control group (*p* ≥ 0.05). These findings are shown in Figure 1a.

#### 3.1.2. Effect of LBP on Feed Intake

The feed intake of mice in the model group was significantly decreased compared with the control group (*p* < 0.01). In the LBP administration group, the feed intake of mice significantly increased (*p* < 0.01) and there was no significant difference with the control group (*p* > 0.05). These findings are shown in Figure 1b.

#### 3.1.3. Effect of LBP on Kidney Index

Compared with the control group, the renal index was significantly higher in the model group (*p* < 0.01). The low-dose and high-dose groups showed a significant decreasing trend compared with the model group (*p* < 0.05), and the middle-dose group showed an extremely significant decreasing trend (*p* < 0.01). These findings are shown in Figure 1c.

#### 3.1.4. Effect of LBP on Renal Function Biomarkers

Compared with the control group, the expression levels of BUN (Figure 1d) and Cre (Figure 1e) in the model group were significantly increased (*p* < 0.01). The LBP administration group had significantly reduced expression levels of BUN and Cre (*p* < 0.01). The medium-dose group had downregulated the levels of BUN and Cre better than the high-dose and low-dose groups.

#### 3.1.5. Effects of LBP on Histopathology

**Figure 2:**
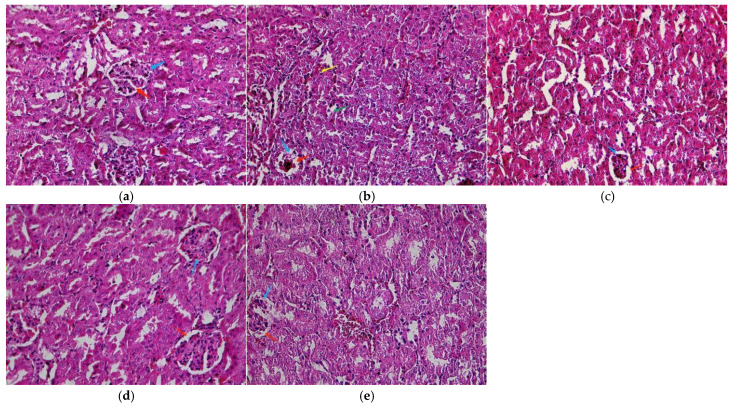
Light micrographs of the kidney cortex. (**a**) Control; (**b**) model; (**c**) low dose; (**d**) middle dose; (**e**) high dose. Glomerulus (blue arrow), urinary space (red arrow), damaged renal tubules with vacuolated cytoplasm (green arrow), and inflammatory cells in the interstitial tissue (yellow arrow). Original magnification: 400.

A normal structure of the renal tissue without histopathological deformations was observed in the control mice (Figure 2a). However, the PbAc_2_-exposed mice had a shrunken and congested glomerulus, widened urinary space, inflammatory cells in the interstitial tissue, and damaged renal tubules with vacuolated cytoplasm (Figure 2b). In contrast, LBP pretreatment alleviated the changes induced in the renal tubules and glomeruli by PbAc_2_. These effects are shown in Table 2.

### 3.2. Mechanism of LBP against Lead-Induced Renal Injury in Mice

#### 3.2.1. Effects of LBP on Oxidative Stress Markers

**Figure 3:**
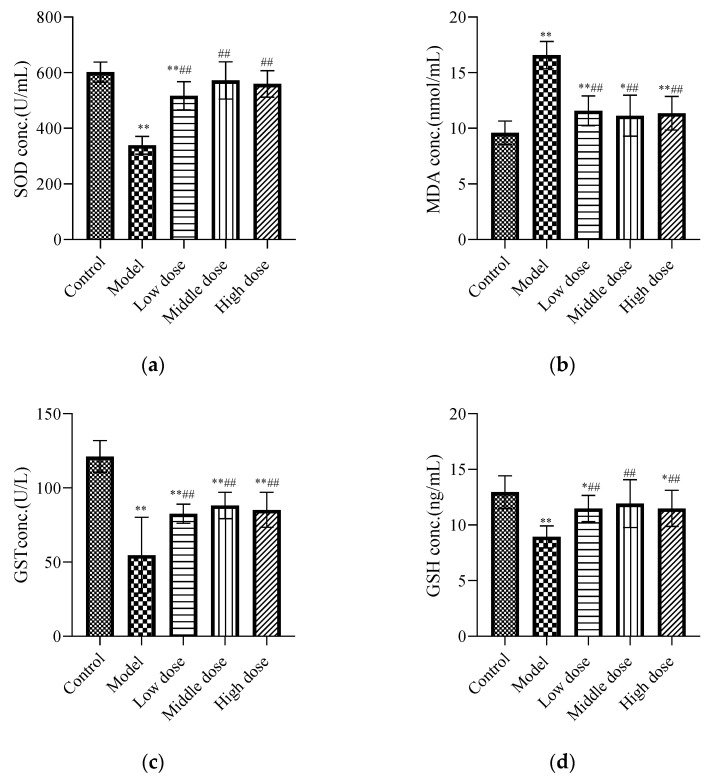
Effects of LBP on oxidative stress markers. The results are expressed as the mean ± SD, *n* = 10. * Statistically significant compared to control group, * *p* < 0.05, ** *p* < 0.01. ^#^ Statistically significant compared to model group, ^##^ *p* < 0.01. (**a**) Expression of superoxide dismutase (SOD); (**b**) Expression of malondialdehyde (MDA); (**c**) Expression of glutathione S-transferase (GST); (**d**) Expression of glutathione (GSH).

The results showed that lead acetate significantly decreased the levels of SOD (Figure 3a), GST (Figure 3c), and GSH (Figure 3d), and significantly increased the level of MDA (Figure 3b) in the kidney tissues of mice (*p* < 0.01), while LBP reversed the alterations in the abovementioned parameters (*p* < 0.01).

#### 3.2.2. Effects of LBP on Renal Inflammatory Biomarkers

**Figure 4:**
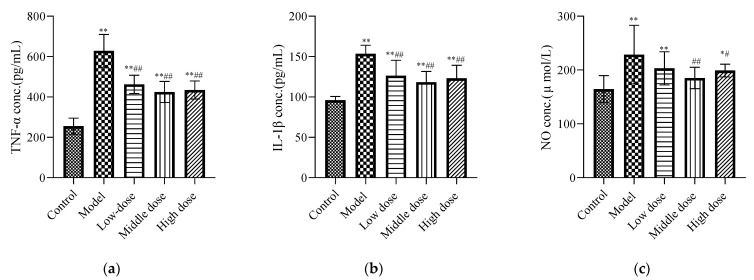
Effects of LBP on renal inflammatory biomarkers. The results are expressed as the mean ± SD, *n* = 10. * Statistically significant compared to control group, * *p* < 0.05, ** *p* < 0.01. ^#^ Statistically significant compared to model group, ^#^ *p* < 0.05, ^##^ *p* < 0.01. (**a**) Expression of tumor necrosis factor-α (TNF-α); (**b**) Expression of interleukin-1β (IL-1β); (**c**) Expression of nitric oxide.

Compared with the control group, the concentrations of IL-1β (Figure 4b), TNF-α (Figure 4a), and NO (Figure 4c) in the model group were increased and showed significant differences (*p* < 0.01). Compared with the model group, the middle-dose group of LBP could downregulate the IL-1β, TNF-α, and NO expression levels, with statistical differences (*p* < 0.01). The high-dose group could significantly downregulate the expression levels of IL-1β and TNF-α (*p* < 0.01), along with the expression level of NO (*p* < 0.05). The LBP low-dose group could significantly downregulate the expression levels of TNF-α and IL-1β with a statistical difference (*p* < 0.01), but the downregulation of NO was not statistically different (*p* > 0.05).

#### 3.2.3. Effects of LBP on Apoptotic Biomarkers

**Figure 5:**
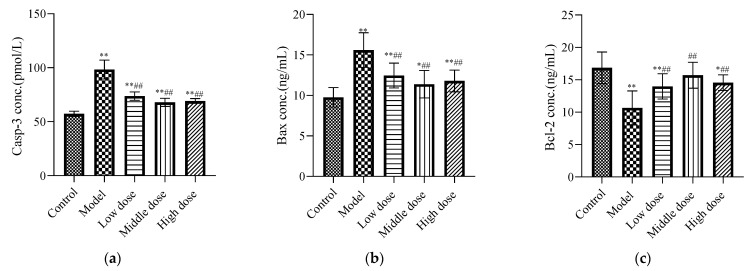
Effects of LBP on apoptotic biomarkers. The results are expressed as the mean ± SD, *n* = 10. * Statistically significant compared to control group, * *p* < 0.05, ** *p* < 0.01. ^#^ Statistically significant compared to model group, ^##^ *p* < 0.01. (**a**) Expression of caspase-3 (Casp-3); (**b**) Expression of Bax; (**c**) Expression of Bcl-2.

Compared with the control group, the expression levels of Casp-3 (Figure 5a) and Bax (Figure 5b) in the model group were significantly increased (*p* < 0.01), while the expression of Bcl-2 (Figure 5c) was significantly decreased (*p* < 0.01). The LBP administration group significantly downregulated Casp-3 and Bax protein expression (*p* < 0.01), and upregulated Bcl-2 protein expression (*p* < 0.01).

#### 3.2.4. Expression of Proteins Related to the Nrf2 Signaling Pathway

**Figure 6:**
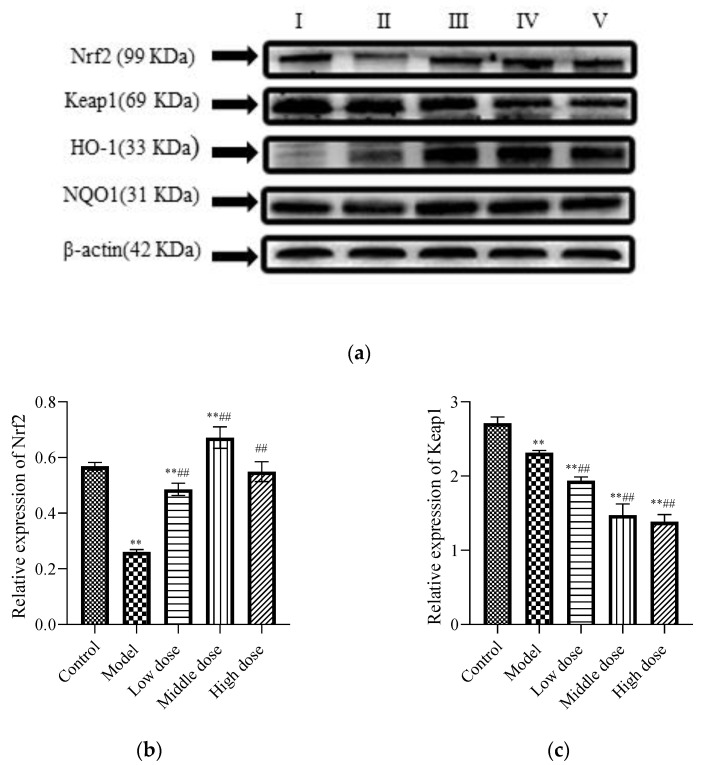

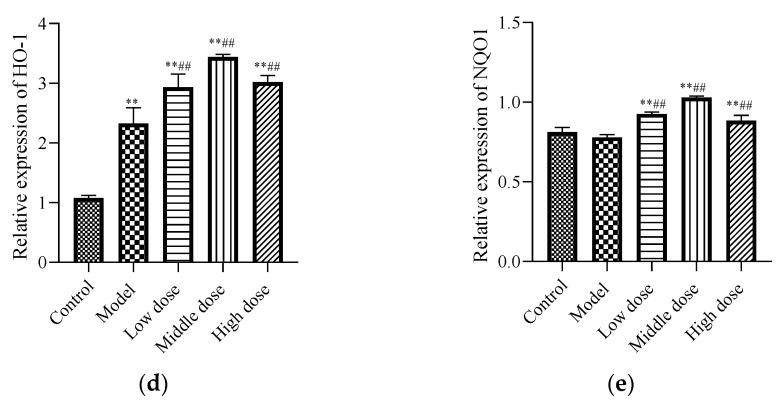
Effects of LBP on Nrf2-related protein expression. I, control; II, model; III, low dose; IV, middle dose; V, high dose. The results are expressed as the mean ± SD, *n* = 10. * Statistically significant compared to control group, ** *p* < 0.01. ^#^ Statistically significant compared to model group, ^##^ *p* < 0.01. (**a**) Western blot analysis of proteins related to nuclear factor erythroid 2-related factor 2 (Nrf2) signaling pathway. (**b**) Expression of Nrf2; (**c**) Expression of Kelch-like ECH-associated protein 1 (Keap1); (**d**) Expression of Oxygenase-1 (HO-1) (**e**) Expression of NAD(P)H dehydrogenase, quinone 1 (NQO1).

To study the signal pathway of LBP against lead-induced renal injury, the expression of Nrf2 signal pathway-related proteins was detected (Figure 6a). The results showed that compared with the control group, the protein expression levels of Nrf2 (Figure 6b) and Keap1 (Figure 6c) in the model group were significantly decreased (*p* < 0.01). Further, the protein expression of HO-1 (Figure 6d) was significantly increased (*p* < 0.01) and the protein expression of NQO1 (Figure 6e) was not statistically different (*p* > 0.05). Compared with the model group, the protein expression levels of Nrf2, HO-1, and NQO1 in the LBP administration group were significantly increased (*p* < 0.01), with the most significant trend observed in the middle-dosage group. All of the Keap1 protein expression levels were significantly decreased (*p* < 0.01). These results indicated that lead could lead to disruption of the antioxidant system by depleting the Nrf2 and Keap1 proteins in vivo, while LBP could activate the Nrf2 signaling pathway and promote the production of antioxidant enzymes protecting against lead-induced kidney injury, with the medium dose having the best effect.

### 3.3. Dose Effect Analysis Results

#### 3.3.1. Multivariate Statistics

**Figure 7:**
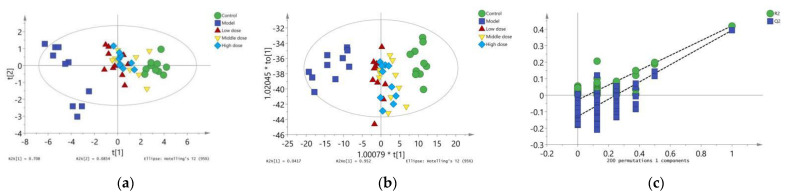
Results of multivariate statistical analysis. (**a**) Principal component analysis diagram; (**b**) Orthogonal partial least squares discriminant analysis diagram; (**c**) Verification diagram of orthogonal partial least squares discriminant analysis.

The PCA results are shown in (Figure 7a). The R2X was 0.794 and Q2 was 0.586, indicating that the PCA model had good interpretation and prediction ability. There was an obvious separation trend between the control group and the model group in PCA score plots, which indicated that lead could induce renal injury in mice through oxidative stress and inflammatory and apoptotic responses. The position of mice in the PCA score plot of the LBP administration group was close to the control group. Mice in the middle-dose group were closer to the control group, which indicated that LBP could protect mice from lead-induced renal injury by inhibiting oxidative stress, inflammation, and apoptosis, with the middle-dose group having the best efficacy.

To further show the variation trend among the groups, OPLS-DA model results showed that the R2X was 0.994, R2Y was 0.23, and Q2 of 200 permutation validation was 21.1%, confirming that this OPLS-DA model had a good fit and predictive ability (Figure 7b). Furthermore, the intercept of the Q2 regression line was −0.123 (Figure 7c), indicating that the model was not overfitted and was statistically significant. Based on these results, the OPLS-DA score showed consistent results with the PCA model, which further illustrated that LBP could protect from lead-induced renal injury by inhibiting oxidative stress, inflammation, and apoptotic responses. The efficacy was better at the middle dose, followed by the high dose, and finally the low dose.

#### 3.3.2. Cluster Heatmap Analysis

**Figure 8:**
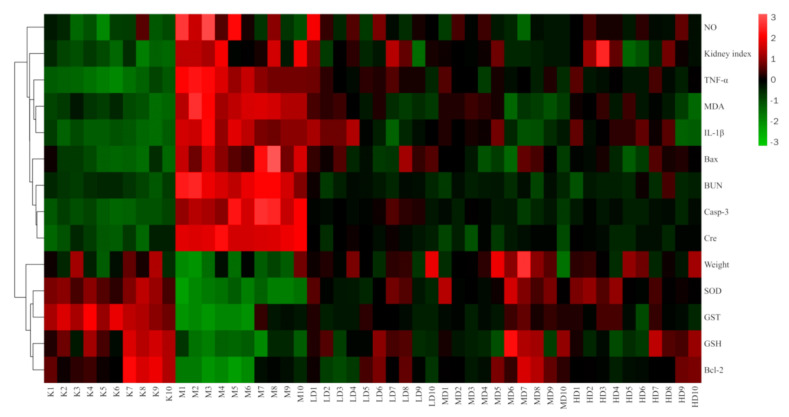
Clustering heatmap results. “*n*” indicates the number of mice, Kn the control group, Mn the model group, LD the low-dose group, MD the medium-dose group, and HD the high-dose group. Nitric oxide (NO); Tumor necrosis factor-α (TNF-α); Malondialdehyde (MDA); Interleukin-1β (IL-1β); Serum creatinine (Cre); Blood urea nitrogen (BUN); Caspase-3 (Casp-3); Superoxide dismutase (SOD); Glutathione S-transferase (GST); Glutathione (GSH).

The results of the cluster heatmap showed that compared with the control group, the indexes of body weight, SOD, GST, GSH, and Bcl-2 in the model group were downregulated, while the NO, kidney index, TNF-α, IL-1β, MDA, Bax, BUN, case-3, and Cre were upregulated. Compared with the model group, the LBP administration groups all presented parameters that reversed the above indicators, and all showed a tendency to recover towards the control group, with the middle dose the closest to the control group. This suggests that LBP can inhibit lead-induced kidney damage through the antioxidant, anti-inflammatory, and anti-apoptotic pathways, with the best effect at medium doses (Figure 8).

## 4. Discussion

It is public knowledge that lead exposure or lead poisoning can cause a series of physiological, biochemical, and behavioral disorders in experimental animals and humans [33,34,35].

Our study showed that the growth rate of mice was significantly slower, and it significantly reduced the body weight of mice after lead exposure (Figure 1a), which was consistent with previous findings [36]. However, supplementation of LBP to PbAc2-treated mice significantly improved the growth rate and average daily feed intake (Figure 1b), and it was able to increase the weights of the mice (Table 1). Early studies have shown that lead exposure causes the body to be immunosuppressed, which increases disease susceptibility [37], and LBP is considered to be a good source of potential prebiotics that can enhance the gut microbiota, increase the level of beneficial bacteria, and regulate innate immune responses [38]. Chen [39] et al.’s study showed that LBP could significantly increase the average daily weight and daily feed intake of weaned piglets. The mechanism may be through enhancing the body’s immune status and antioxidant capacity and improving the intestinal microbial population. It is suggested that LBP may enhance the immunity and antioxidant capacity of the body, improve the number of intestinal microflora, enhance the appetite of lead poisoned mice, and promote the growth of mice.

A large number of studies have shown that lead exposure not only produces weight loss in mice but also induces multiple organ injuries in humans. When exposed to lead, the kidney is one of the most affected organs [40]. Our results showed that the kidney index was significantly increased in the model group compared with the control group (Figure 1c), which was consistent with previous reports [7], and LBP administration reduced the kidney index of mice with lead toxicity. Lead-induced kidney injury was also confirmed by the observation of renal pathological sections (Figure 2), which showed obvious early-stage proximal tubule vacuolization, tissue inflammation, and tubule dilatation in the kidneys of model mice compared with controls, whereas the mice in the LBP administration group all improved in terms of the above symptoms. Researchers found that some natural compounds can reduce lead acetate-induced nephrotoxicity through antioxidant, anti-inflammatory, and anti-apoptotic activities [6,41]. LBP has been proved to possess biological properties such as antioxidant, anti-inflammation, and anti-apoptosis properties in different models, tissues, and organs. Huang et al. [30] found that LBP could reduce inflammation and activate antioxidant responses to protect from kidney injury in septic rats by regulating proinflammatory cytokine levels and the Keap1-Nrf2/antioxidant response element(ARE) signaling pathways. Xiao et al.’s [42] experiment showed that LBP alleviated ethanol-induced liver injury by reducing hepatic apoptosis, oxidative stress, and NOD-like receptor 3 (NLRP3) inflammasome.

BUN and Cre levels are commonly used to reflect renal function status. Both are nitrogen-containing endproducts of metabolism; BUN is the primary metabolite derived from dietary protein and tissue protein turnover, and Cre is a product of muscle creatine catabolism [43]. Our study showed that the levels of BUN (Figure 1d) and Cre (Figure 1e) were significantly increased in the model mice compared with the control group, which indicated that our modeling was successful and that the LBP-administrated group exhibited significantly lower levels of BUN and Cre than the model group, suggesting that LBP may have blunted the development of renal injury. According to Zhao et al. [44], LBP can improve the renal function of diabetic rats and reduce kidney damage by reducing oxidative stress and inhibiting the activity of extracellular signal-regulated kinases 1 and 2 (ERK1/2) in mesangial cells. Du et al. [45]. believed that the mechanism of LBP protecting the kidneys of diabetic rats was the inhibition of proteinuria, the blood urea nitrogen concentration, and serum inflammatory factors (including IL-2, IL-6, and TNF-α). Yu et al.’s [46] study showed that LBP has renoprotective effects, which may protect mice from hyperuricemia by promoting renal uric acid excretion. Liao et al. [47] suggested that the mechanism of LBP against high-fat diet-induced renal injury in rats may be to mediate lipid metabolism, enhance anti-inflammatory responses, and ameliorate renal injury caused by lipid metabolism regulators.

Based on the above conclusions, the mechanism of LBP’s protection against lead-induced renal injury was studied. It is well-known that the mechanism of lead-induced kidney injury is multifaceted. Lead can stop the synthesis of structural proteins by binding to sulfhydryl proteins, thereby reducing the reserve of the organism’s sulfhydryl antioxidants, and eventually causing damage to the organism by depleting the body’s antioxidants [48,49,50]. GSH is an intracellular non-enzymatic antioxidant-containing thiols that have antioxidant and detoxification effects [51]. Therefore, lead can inhibit the activity of antioxidant GSH by inhibiting the effect of thiols [52]. As mentioned in the article by Veena Sharma et al. [53], peroxyl radicals are generated after lead poisoning, endoperoxides are generated through cyclization reaction, lipid peroxidation is stimulated, and the increased level of MDA indicates enhanced lipid peroxidation. Lead can induce oxidative stress (OS) by producing reactive oxygen species (ROS). The production of reactive oxygen species exceeds the ability of the antioxidant system to protect cells from oxidative molecules, and so oxidative stress will occur [54]. SOD provides the first line of defense against free radicals by making toxic superoxide into less toxic H_2_O_2_ [55], while GSH and GST generally guarantee the removal of H_2_O_2_. Our experimental results confirmed the above conclusion that compared with the control group, the levels of GST (Figure 3c), GSH (Figure 3d), and SOD (Figure 3a) in the model group significantly decreased, and the level of MDA (Figure 3b) significantly increased. It was suggested that lead acetate caused oxidative stress and lipid peroxidation in mice. Compared with the model group, the LBP administration group significantly upregulated the levels of GST, GSH, and SOD and downregulated the level of MDA in mice with lead-induced nephrotoxicity. Experiments by Varoni et al. [56] demonstrated that LBP could inhibit the oxidative stress response and thus protect from cadmium-induced rat liver damage. The experiment of Pan et al. showed that [57] LBP can improve retinal oxidative stress and exert beneficial neuroprotective effects in diabetic rats, and its mechanism may be associated with the activation of the Nrf2/HO-1 antioxidant pathway. It is suggested that LBP may regulate the production of antioxidant enzymes against lead-induced renal injury by activating the Nrf2 signaling pathway.

Lead-induced cellular inflammation is also considered to be one of the mechanisms of renal injury [41]. Therefore, the inflammatory markers in the kidney tissue of each group of mice were detected. TNF-α and IL-1β are produced from the activated macrophages and lymphocytes; they are involved in the local and systemic inflammatory responses at the injured site [58]. Moreover, during this inflammatory response, ROS/nitrogenous species (RNS) are produced, which can lead to obvious damage to tissues and cells [35]. Our study showed that the levels of IL-1β (Figure 4b), TNF-α (Figure 4a), and NO (Figure 4c) were increased in the model group compared with the control group. Excitingly, the LBP administration group could significantly downregulate the concentrations of TNF-α, NO, and IL-1β in lead-induced nephrotoxic mice. According to Xiong et al. [23], LBP reduced inflammation by upregulating nuclear factor erythroid-2-related factor 2 (Nrf2) and oxygenase-1 (HO-1). It is suggested that LBP may protect the kidneys of lead-induced nephrotoxic mice by inhibiting the expression of proinflammatory cytokines such as IL-1β, TNF-α, and NO and the activation of the Nrf2 pathway.

Renal apoptosis is associated with the development of oxidative stress and inflammation [59]. Therefore, apoptosis markers were detected. Our results showed that the concentrations of the Casp-3 (Figure 5a) and Bax (Figure 5b) proteins were significantly increased, while the concentrations of Bcl-2 (Figure 5c) protein were significantly decreased in the model group compared with the control group. This is consistent with the results of previous studies [6,52]. Bax is a principal apoptotic protein, whereas Bcl-2 is an apoptosis antagonist [60]. The best recognized biochemical hallmark of both the early and late stages of apoptosis is the activation of cysteine proteases (caspases). Detection of active caspase-3 in cells and tissues is an important method for apoptosis induced by a wide variety of apoptotic signals [61]. Liu et al. suggested that lead can induce depletion of mitochondrial membrane potential and then cause apoptosis in the proximal tubules of rats [12]. Additionally, it has been reported in the literature that lead-induced toxicity is associated with infiltration of inflammatory cells in the interstitial space, which stimulates inflammation-mediated apoptosis via the extrinsic pathway [62]. Strikingly, our results showed that LBP significantly downregulated Casp-3 enzyme and Bax protein concentrations, while upregulating Bcl-2 protein concentrations in the kidney tissues of lead-toxic mice. Research by Yu et al. showed [63] that LBP could protect primary cultured hippocampal neurons from oxygen-glucose deprivation/reoxygenation-induced apoptosis and autophagy cell death through phosphoinositide 3 kinase (PI3K)/protein kinase B (Akt)/mammalian target of the rapamycin (mTOR) signaling pathway. Yang et al. [21] showed that LBP could promote the development of pre-cryopreserved mouse two-cell embryos by restoring the mitochondrial function and downregulating the production of reactive oxygen species. In addition, the research of Luo et al. [64] showed that LBP could prevent the decline of mitochondrial membrane potential, upregulate the expression of Bcl-2, and downregulate the expression of Bax, thereby reducing the apoptosis of spermatogenic cells. These results suggest that LBP may protect renal cells from lead-induced apoptosis by improving mitochondrial dysfunction, upregulating the expression of Bcl-2, and downregulating the expression of Bax and Casp-3.

The Nrf2 signaling pathway plays a key role in maintaining cell homeostasis under conditions of oxidative stress, inflammation, apoptosis, and cancer [65]. By analyzing the expression of oxidative stress markers, inflammatory markers, and apoptosis markers in the kidneys of mice, it is speculated that the Nrf2 signal pathway may be one of the pharmacological targets of LBP against lead-induced renal injury. Therefore, expressions of Nrf2 signaling pathway-related proteins in the kidneys of mice were detected. Under normal physiological circumstances, Nrf2 is anchored in the cytoplasm by Keap1, which as an action substrate of the Cullin 3 (Cul3)-dependent E3 ubiquitin ligase complex, could promote Nrf2 to be ubiquitinated and rapidly degraded by the proteasome [66]. However, when the cell is attacked by ROS or electrophiles, Nrf2 is dissociated from Keap1 and rapidly translocated into the nucleus. It forms a heterodimer with the small Maf protein and then combines with antioxidant response elements to transcriptively activate the expression of antioxidant factor genes such as GSH, GST, SOD, NQO1, and HO-1, regulated by Nrf2 [65]. However, this is not always the case. The experiments of Liu et al. [67] and Liu et al. [68] showed that lead inhibited the expression of Nrf2 signaling-related proteins and then induced oxidative stress, inflammation, and apoptosis. Interestingly, our experimental results are not entirely consistent with previous studies. Our results showed that lead exposure significantly downregulated Nrf2 (Figure 6b) and keap1 (Figure 6c) expressions and significantly upregulated HO-1 (Figure 6d) expression, while NQO1 (Figure 6e) expression was not significantly different. This indicates that lead inhibits the activation of the Nfr2 signaling pathway. It is well known that the mechanism of lead-induced renal injury is multifaceted, and pathways such as mitogen-activated protein kinase (MAPK), Akt/protein kinase B (PKB) can all regulate HO-1 expression [69]. Liu et al. [70] have found that lead-induced ROS can promote the MAPK pathway, thus promoting its downstream pathway, and inducing abnormal release of inflammatory factors, leading to inflammation and kidney damage. Therefore, we speculated that the lead-induced upregulation of HO-1 protein in this experiment might be induced by other pathways rather than by the activation of the Nrf2 signaling pathway.

Consistent with our hypothesis, LBP significantly downregulated the expression of keap1 (Figure 6c), resulting in the loss of Nrf2 control, activated Nrf2 (Figure 6b) translocation to the nucleus, and upregulated the expression of downstream antioxidant factors, thus alleviating oxidative stress, inflammation, and apoptosis of renal tissue. Notably, the decrease in keap1 in this experiment seemed to have a dose-dependent relationship with LBP, but the upregulation of Nrf2 expression by LBP was most significant in the medium-dose group. It is well known that the activation of the Nrf2 signaling pathway is multipathway [71], and the targets and efficacy of different doses of drugs are various [72]. Zheng et al.’ s experiment showed [73] that LBP could mediate Nrf2 activation by the AMP-activated protein kinase (AMPK) pathway and then alleviate acute lung injury induced by hyperoxia in mice. It is suggested that LBP activates the Nrf2 signaling pathway and resists lead-induced renal damage may be mediated by multiple pathways, and the targets and efficacy of the Nrf2 signaling pathway mediated by different doses of LBP are not completely the same. In a word, LBP activates the Nrf2 signaling pathway to resist lead-induced kidney injury, which is at least related to the downregulation of keap1 by LBP, and the activation Nrf2 effect of the medium dose is the best.

Finally, the dose–effect relationship of LBP against lead-induced renal injury was analyzed by establishing PCA (Figure 7a) and OPLS-DA (Figure 7b) models and a clustering heatmap (Figure 8). The results showed that the efficacy of LBP in the medium-dose group was better than that in the high-dose group and low-dose group. As a kind of highly polar macromolecule, polysaccharides are widely considered to be poorly absorbed orally [74]. The medium dose of LBP had better efficacy against lead-induced renal injury, which may have been related to the superior bioavailability of the medium dose of LBP.

## 5. Conclusions

**Figure 9:**
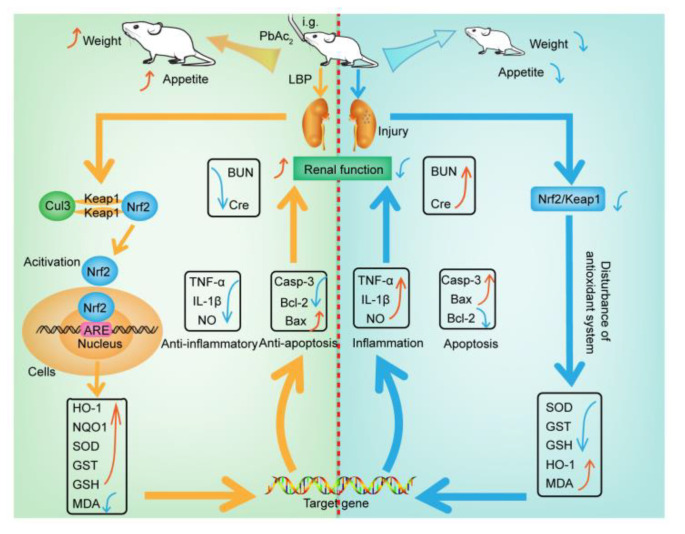
The mechanism of lead-induced renal injury in mice and the mechanism of LBP against lead-induced renal injury. Intragastric administration (i.g); Lead acetate (PbAc_2_); Nitric oxide (NO); Tumor necrosis factor-α (TNF-α); Malondialdehyde (MDA); Interleukin-1β (IL-1β); Serum creatinine (Cre); Blood urea nitrogen (BUN); Caspase-3 (Casp-3); Superoxide dismutase (SOD); Glutathione S-transferase (GST); Glutathione (GSH); Cullin 3 (Cul3); Nuclear factor erythroid 2-related factor 2 (Nrf2); Kelch-like ECH-associated protein 1 (Keap1); Oxygenase-1 (HO-1); NAD(P)H dehydrogenase, quinone 1 (NQO1); *Lycium barbarum* polysaccharide (LBP); Antioxidant response element(ARE); Indicates up-regulation or recovery (↑); Indicates downregulation or inhibition (↓).

This study aimed to investigate the efficacy and mechanism of LBP against lead-induced renal injury. The results of the pharmacodynamics experiment showed that LBP could significantly restore the renal function and renal pathological damage of mice exposed to lead toxicity, as well as increasing the food intake of mice exposed to lead toxicity and promoting their growth. Dose effect results indicated that a medium dose of LBP was superior to high and low doses. The results of mechanistic experiments showed that LBP could attenuate oxidative stress, inflammation, and apoptosis in the kidneys of mice exposed to lead toxicity by activating the Nrf2 signaling pathway. The efficacy and mechanism of LBP against lead-induced renal injury are shown in Figure 9.

## Figures and Tables

**Table 1:** Initial weight and final weight as the mean ± SD (*n* = 10).

Group	Initial Weight (g)	Final Weight (g)
Control	18.79 ± 1.21	33.40 ± 2.33
Model	18.59 ± 0.64	30.26 ± 2.64 *
Low dose	18.68 ± 0.99	33.99 ± 2.13 ^##^
Middle dose	18.77 ± 1.01	34.87 ± 3.51 ^##^
High dose	19.14 ± 1.27	34.00 ± 1.79 ^##^

* Statistically significant compared to control group, * *p* < 0.05. ^#^ Statistically significant compared to model group, ^##^ *p* < 0.01.

**Table 2:** Grading of histopathological changes in kidney.

Group	Pathological Changes of Glomerulus	Renal Tubular Injury	Inflammation
Control	-	-	-
Model	+++	++	+++
Low dose	++	+	+
Middle dose	+	+	+
High dose	+	+	+

Note: +, mild; ++, moderate; and +++, severe histopathological changes.

## Data Availability

The data presented in this study are available on request from the corresponding author.

## References

[1] Gloaguen T.V., Motta P.N.S.D., Couto C.F. (2020). A grain-size correction for metal pollution indexes in river sediments. Int. J. Sediment Res..

[2] Hou S., Zheng N., Tang L., Ji X., Li Y., Hua X. (2019). Pollution characteristics, sources, and health risk assessment of human exposure to Cu, Zn, Cd and Pb pollution in urban street dust across China between 2009 and 2018. Environ. Int..

[3] Shi T., Ma J., Zhang Y., Liu C., Zhao L. (2019). Status of lead accumulation in agricultural soils across China (1979–2016). Environ. Int..

[4] Abdel Moneim A.E., Dkhil M.A., Al-Quraishy S. (2011). The protective effect of flaxseed oil on lead acetate-induced renal toxicity in rats. J. Hazard. Mater..

[5] Sauser L., Shoshan M.S. (2020). Harnessing Peptides against lead pollution and poisoning: Achievements and prospects. J. Inorg. Biochem..

[6] Albarakati A.J.A., Baty R.S., Aljoudi A.M., Habotta O.A., Elmahallawy E.K., Kassab R.B., Abdel Moneim A.E. (2020). Luteolin protects against lead acetate-induced nephrotoxicity through antioxidant, anti-inflammatory, anti-apoptotic, and Nrf2/HO-1 signaling pathways. Mol. Biol. Rep..

[7] Zhang Z., Gao X., Guo M., Jiang H., Cao Y., Zhang N. (2017). The protective effect of baicalin against lead-induced renal oxidative damage in mice. Biol. Trace Elem. Res..

[8] Alhusaini A.M., Fadda L.M., Hasan I.H., Ali H.M., Badr A., Elorabi N., Alomar H., Alqahtani Q., Zakaria E., Alanazi A. (2020). Role of some natural anti-oxidants in the down regulation of Kim, VCAM1, Cystatin C protein expression in lead acetate-induced acute kidney injury. Pharmacol. Rep. PR.

[9] Fan Y., Zhao X., Yu J., Xie J., Li C., Liu D., Tang C., Wang C. (2020). Lead-induced oxidative damage in rats/mice: A meta-analysis. J. Trace Elem. Med. Biol. Organ Soc. Miner. Trace Elem..

[10] Hasanein P., Riahi H. (2018). Preventive use of berberine in inhibition of lead-induced renal injury in rats. Environ. Sci. Pollut. Res. Int..

[11] Wang Z.K., Zhou X.L., Song X.B., Zhuang D.M., Wang Z.Y., Yang D.B., Wang L. (2016). Alleviation of lead-induced apoptosis by puerarin via inhibiting mitochondrial permeability transition pore opening in primary cultures of rat proximal tubular cells. Biol. Trace Elem. Res..

[12] Liu G., Wang Z.K., Wang Z.Y., Yang D.B., Liu Z.P., Wang L. (2016). Mitochondrial permeability transition and its regulatory components are implicated in apoptosis of primary cultures of rat proximal tubular cells exposed to lead. Arch. Toxicol..

[13] Amadi C.N., Offor S.J., Frazzoli C., Orisakwe O.E. (2019). Natural antidotes and management of metal toxicity. Environ. Sci. Pollut. Res. Int..

[14] Flora S.J., Pachauri V. (2010). Chelation in metal intoxication. Int. J. Environ. Res. Public Health.

[15] Bradberry S., Vale A. (2009). A comparison of sodium calcium edetate (edetate calcium disodium) and succimer (DMSA) in the treatment of inorganic lead poisoning. Clin. Toxicol..

[16] El-Boshy M.E., Refaat B., Qasem A.H., Khan A., Ghaith M., Almasmoum H., Mahbub A., Almaimani R.A. (2019). The remedial effect of Thymus vulgaris extract against lead toxicity-induced oxidative stress, hepatorenal damage, immunosuppression, and hematological disorders in rats. Environ. Sci. Pollut. Res. Int..

[17] Soussi A., Gargouri M., El Feki A. (2018). Potential immunomodulatory and antioxidant effects of walnut Juglans regia vegetable oil against lead-mediated hepatic damage and their interaction with lipase activity in rats. Environ. Toxicol..

[18] El-Boshy M., Ashshi A., Gaith M., Qusty N., Bokhary T., AlTaweel N., Abdelhady M. (2017). Studies on the protective effect of the artichoke (Cynara scolymus) leaf extract against cadmium toxicity-induced oxidative stress, hepatorenal damage, and immunosuppressive and hematological disorders in rats. Environ. Sci. Pollut. Res. Int..

[19] Yao R., Heinrich M., Weckerle C.S. (2018). The genus Lycium as food and medicine: A botanical, ethnobotanical and historical review. J. Ethnopharmacol..

[20] Wang W., Liu M., Wang Y., Yang T., Li D., Ding F., Sun H., Bai G., Li Q. (2018). Lycium barbarum polysaccharide promotes maturation of dendritic cell via notch signaling and strengthens dendritic cell mediated t lymphocyte cytotoxicity on colon cancer cell CT26-WT. Evid.-Based Complement. Altern. Med..

[21] Yang L., Gao Z., Lei L., Lv Q., Zhao Q., Li L., Cao X., Fu W. (2019). Lycium barbarum polysaccharide enhances development of previously-cryopreserved murine two-cell embryos via restoration of mitochondrial function and down-regulated generation of reactive oxygen species. J. Reprod. Dev..

[22] Zhang Z., Zhou Y., Fan H., Billy K.J., Zhao Y., Zhan X., Yang L., Jia Y. (2019). Effects of Lycium barbarum polysaccharides on health and aging of C. elegans depend on daf-12/daf-16. Oxidative Med. Cell. Longev..

[23] Xiong G.F., Li D.W., Zheng M.B., Liu S.C. (2019). The effects of Lycium Barbarum polysaccharide (LBP) in a mouse model of cerulein-induced acute pancreatitis. Med. Sci. Monit..

[24] Cai H., Liu F., Zuo P., Huang G., Song Z., Wang T., Lu H., Guo F., Han C., Sun G. (2015). Practical Application of antidiabetic efficacy of Lycium barbarum polysaccharide in patients with type 2 diabetes. Med. Chem..

[25] Luo Q., Cai Y., Yan J., Sun M., Corke H. (2004). Hypoglycemic and hypolipidemic effects and antioxidant activity of fruit extracts from Lycium barbarum. Life Sci..

[26] Zhou Y., Duan Y., Huang S., Zhou X., Zhou L., Hu T., Yang Y., Lu J., Ding K., Guo D. (2020). Polysaccharides from Lycium barbarum ameliorate amyloid pathology and cognitive functions in APP/PS1 transgenic mice. Int. J. Biol. Macromol..

[27] Yang F., Wei Y., Liao B., Wei G., Qin H., Pang X., Wang J. (2018). Lycium barbarum polysaccharide prevents cisplatin-induced MLTC-1 cell apoptosis and autophagy via regulating endoplasmic reticulum stress pathway. Drug Des. Dev. Ther..

[28] Lakshmanan Y., Wong F.S.Y., Zuo B., So K.F., Bui B.V., Chan H.H. (2019). Posttreatment intervention with Lycium Barbarum polysaccharides is neuroprotective in a rat model of chronic ocular hypertension. Investig. Ophthalmol. Vis. Sci..

[29] Ding Y., Yan Y., Chen D., Ran L., Mi J., Lu L., Jing B., Li X., Zeng X., Cao Y. (2019). Modulating effects of polysaccharides from the fruits of Lycium barbarum on the immune response and gut microbiota in cyclophosphamide-treated mice. Food Funct..

[30] Huang Y., Zhou F., Shen C., Wang H., Xiao Y. (2019). LBP reduces the inflammatory injury of kidney in septic rat and regulates the Keap1-Nrf2/ARE signaling pathway1. Acta Cir. Bras..

[31] Abdel-Moneim A.M., El-Toweissy M.Y., Ali A.M., Awad Allah A.A., Darwish H.S., Sadek I.A. (2015). Curcumin ameliorates lead (Pb(2+))-induced hemato-biochemical alterations and renal oxidative damage in a rat model. Biol. Trace Elem. Res..

[32] Worley B., Halouska S., Powers R. (2012). Utilities for quantifying separation in PCA/PLS-DA scores plots. Anal. Biochem..

[33] Ibrahim N.M., Eweis E.A., El-Beltagi H.S., Abdel-Mobdy Y.E. (2012). Effect of lead acetate toxicity on experimental male albino rat. Asian Pac. J. Trop. Biomed..

[34] Abdou H.M., Hassan M.A. (2014). Protective role of omega-3 polyunsaturated fatty acid against lead acetate-induced toxicity in liver and kidney of female rats. BioMed Res. Int..

[35] Shi Y., Tian C., Yu X., Fang Y., Zhao X., Zhang X., Xia D. (2020). Protective effects of smilax glabra Roxb. against lead-induced renal oxidative stress, inflammation and apoptosis in weaning rats and HEK-293 cells. Front. Pharmacol..

[36] Daku A.B., Mustapha S., Salisu A.I., El-Ta’alu A.B. (2019). Age-related effects of lead poisoning on some haematological parameters in adult wistar rats. Niger. J. Physiol. Sci. Off. Publ. Physiol. Soc. Niger..

[37] Xu X.H., Meng X., Gan H.T., Liu T.H., Yao H.Y., Zhu X.Y., Xu G.C., Xu J.T. (2019). Immune response, MT and HSP70 gene expression, and bioaccumulation induced by lead exposure of the marine crab, Charybdis japonica. Aquat. Toxicol..

[38] Zhu W., Zhou S., Liu J., McLean R.J.C., Chu W. (2020). Prebiotic, immuno-stimulating and gut microbiota-modulating effects of Lycium barbarum polysaccharide. Biomed. Pharmacother..

[39] Chen J., Long L., Jiang Q., Kang B., Li Y., Yin J. (2020). Effects of dietary supplementation of Lycium barbarum polysaccharides on growth performance, immune status, antioxidant capacity and selected microbial populations of weaned piglets. J. Anim. Physiol. Anim. Nutr..

[40] Agrawal S., Flora G., Bhatnagar P., Flora S.J. (2014). Comparative oxidative stress, metallothionein induction and organ toxicity following chronic exposure to arsenic, lead and mercury in rats. Cell. Mol. Biol..

[41] Abdel-Daim M.M., Alkahtani S., Almeer R., Albasher G. (2020). Alleviation of lead acetate-induced nephrotoxicity by Moringa oleifera extract in rats: Highlighting the antioxidant, anti-inflammatory, and anti-apoptotic activities. Environ. Sci. Pollut. Res. Int..

[42] Xiao J., Zhu Y., Liu Y., Tipoe G.L., Xing F., So K.F. (2014). Lycium barbarum polysaccharide attenuates alcoholic cellular injury through TXNIP-NLRP3 inflammasome pathway. Int. J. Biol. Macromol..

[43] Hosten A.O., Walker H.K., Hall W.D., Hurst J.W. (1990). BUN and Creatinine. Clinical Methods: The History, Physical, and Laboratory Examinations.

[44] Zhao R., Li Q.W., Li J., Zhang T. (2009). Protective effect of Lycium barbarum polysaccharide 4 on kidneys in streptozotocin-induced diabetic rats. Can. J. Physiol. Pharmacol..

[45] Du M., Hu X., Kou L., Zhang B., Zhang C. (2016). Lycium barbarum polysaccharide mediated the antidiabetic and antinephritic effects in diet-streptozotocin-induced diabetic Sprague Dawley rats via regulation of NF-κB. BioMed Res. Int..

[46] Yu X., Zhang L., Zhang P., Zhi J., Xing R., He L. (2020). Lycium barbarum polysaccharides protect mice from hyperuricaemia through promoting kidney excretion of uric acid and inhibiting liver xanthine oxidase. Pharm. Biol..

[47] Liao J., Liu B., Zhong W., Wang G.D., Xu Y.L., Chen X. (2019). Protective effect of Lycium barbarum polysaccharides against high-fat diet-induced renal injury and lipid deposition in rat kidneys. J. Biol. Regul. Homeost. Agents.

[48] Wu X., Cobbina S.J., Mao G., Xu H., Zhang Z., Yang L. (2016). A review of toxicity and mechanisms of individual and mixtures of heavy metals in the environment. Environ. Sci. Pollut. Res. Int..

[49] Ercal N., Gurer-Orhan H., Aykin-Burns N. (2001). Toxic metals and oxidative stress part I: Mechanisms involved in metal-induced oxidative damage. Curr. Top. Med. Chem..

[50] Patrick L. (2006). Lead toxicity part II: The role of free radical damage and the use of antioxidants in the pathology and treatment of lead toxicity. Altern. Med. Rev. J. Clin. Ther..

[51] Oyagbemi A.A., Omobowale T.O., Akinrinde A.S., Saba A.B., Ogunpolu B.S., Daramola O. (2015). Lack of reversal of oxidative damage in renal tissues of lead acetate-treated rats. Environ. Toxicol..

[52] Liu C.M., Ma J.Q., Sun Y.Z. (2010). Quercetin protects the rat kidney against oxidative stress-mediated DNA damage and apoptosis induced by lead. Environ. Toxicol. Pharmacol..

[53] Sharma V., Sharma A., Kansal L. (2010). The effect of oral administration of Allium sativum extracts on lead nitrate induced toxicity in male mice. Food Chem. Toxicol. Int. J. Publ. Br. Ind. Biol. Res. Assoc..

[54] Lopes A.C., Peixe T.S., Mesas A.E., Paoliello M.M. (2016). Lead exposure and oxidative stress: A systematic review. Rev. Environ. Contam. Toxicol..

[55] Sakeran M.I., Zidan N., Rehman H., Aziz A.T., Saggu S. (2014). Abrogation by Trifolium alexandrinum root extract on hepatotoxicity induced by acetaminophen in rats. Redox Rep. Commun. Free Radic. Res..

[56] Varoni M.V., Pasciu V., Gadau S.D., Baralla E., Serra E., Palomba D., Demontis M.P. (2017). Possible antioxidant effect of Lycium barbarum polysaccharides on hepatic cadmium-induced oxidative stress in rats. Environ. Sci. Pollut. Res. Int..

[57] Pan H., Shi Z., Yang T.G., Yu L.M., Xu A.L. (2019). The protective effects of lycium barbarum polysaccharides on retinal neurons in diabetic rats and its mechanism. Chin. J. Appl. Physiol..

[58] Albasher G., Albrahim T., Alsultan N., Alfaraj S., Alharthi M.S., Kassab R.B., Abdel Moneim A.E. (2020). Red beetroot extract mitigates chlorpyrifos-induced reprotoxicity associated with oxidative stress, inflammation, and apoptosis in rats. Environ. Sci. Pollut. Res. Int..

[59] Yin M., Jiang N., Guo L., Ni Z., Al-Brakati A.Y., Othman M.S., Abdel Moneim A.E., Kassab R.B. (2019). Oleuropein suppresses oxidative, inflammatory, and apoptotic responses following glycerol-induced acute kidney injury in rats. Life Sci..

[60] Yan Y., Jun C., Lu Y., Jiangmei S. (2019). Combination of metformin and luteolin synergistically protects carbon tetrachloride-induced hepatotoxicity: Mechanism involves antioxidant, anti-inflammatory, antiapoptotic, and Nrf2/HO-1 signaling pathway. BioFactors.

[61] Choudhary G.S., Al-Harbi S., Almasan A. (2015). Caspase-3 activation is a critical determinant of genotoxic stress-induced apoptosis. Methods Mol. Biol..

[62] Metryka E., Chibowska K., Gutowska I., Falkowska A., Kupnicka P., Barczak K., Chlubek D., Baranowska-Bosiacka I. (2018). Lead (Pb) exposure enhances expression of factors associated with inflammation. Int. J. Mol. Sci..

[63] Yu Y., Wu X., Pu J., Luo P., Ma W., Wang J., Wei J., Wang Y., Fei Z. (2018). Lycium barbarum polysaccharide protects against oxygen glucose deprivation/reoxygenation-induced apoptosis and autophagic cell death via the PI3K/Akt/mTOR signaling pathway in primary cultured hippocampal neurons. Biochem. Biophys. Res. Commun..

[64] Luo Q., Li J., Cui X., Yan J., Zhao Q., Xiang C. (2014). The effect of Lycium barbarum polysaccharides on the male rats’ reproductive system and spermatogenic cell apoptosis exposed to low-dose ionizing irradiation. J. Ethnopharmacol..

[65] Tu W., Wang H., Li S., Liu Q., Sha H. (2019). The anti-inflammatory and anti-oxidant mechanisms of the keap1/Nrf2/ARE signaling pathway in chronic diseases. Aging Dis..

[66] Bellezza I., Giambanco I., Minelli A., Donato R. (2018). Nrf2-Keap1 signaling in oxidative and reductive stress. Biochim. Biophys. Acta Mol. Cell Res..

[67] Liu C.M., Tian Z.K., Zhang Y.J., Ming Q.L., Ma J.Q., Ji L.P. (2020). Effects of gastrodin against lead-induced brain injury in mice associated with the Wnt/Nrf2 pathway. Nutrients.

[68] Liu B., Zhang H., Tan X., Yang D., Lv Z., Jiang H., Lu J., Baiyun R., Zhang Z. (2017). GSPE reduces lead-induced oxidative stress by activating the Nrf2 pathway and suppressing miR153 and GSK-3β in rat kidney. Oncotarget.

[69] Waza A.A., Hamid Z., Ali S., Bhat S.A., Bhat M.A. (2018). A review on heme oxygenase-1 induction: Is it a necessary evil. Inflamm. Res. Off. J. Eur. Histamine Res. Soc..

[70] Liu C.M., Sun Y.Z., Sun J.M., Ma J.Q., Cheng C. (2012). Protective role of quercetin against lead-induced inflammatory response in rat kidney through the ROS-mediated MAPKs and NF-κB pathway. Biochim. Biophys. Acta.

[71] Tonelli C., Chio I.I.C., Tuveson D.A. (2018). Transcriptional regulation by Nrf2. Antioxid. Redox Signal..

[72] Wang Q., Qian S.Y., Yang X.L., Jin J.Y., Li Y.Q. (2006). Effects of different doses of hydrocortisone on the disorder of coagulation in rats at early stage of septic shock. Chin. J. Pediatr..

[73] Zheng G., Ren H., Li H., Li X., Dong T., Xu S., Yan Y., Sun B., Bai J., Li Y. (2019). Lycium barbarum polysaccharide reduces hyperoxic acute lung injury in mice through Nrf2 pathway. Biomed. Pharmacother..

[74] Li L., Yao H., Li X., Zhang Q., Wu X., Wong T., Zheng H., Fung H., Yang B., Ma D. (2019). Destiny of Dendrobium officinale polysaccharide after oral administration: Indigestible and nonabsorbing, ends in modulating gut microbiota. J. Agric. Food Chem..

